# Diabetes-Related Health Care Utilization and Dietary Intake Among Food Pantry Clients

**DOI:** 10.1089/heq.2019.0102

**Published:** 2019-12-17

**Authors:** Eric M. Bomberg, Sophie Rosenmoss, Morgan Smith, Elaine Waxman, Hilary K. Seligman

**Affiliations:** ^1^ Department of Pediatrics, Division of Endocrinology and the Center for Pediatric Obesity Medicine, University of Minnesota, Minneapolis, Minnesota.; ^2^ Center for Vulnerable Populations at Zuckerberg San Francisco General Hospital, San Francisco, California.; ^3^ Feeding America, Chicago, Illinois.; ^4^ Urban Institute, Washington, District of Columbia.; ^5^ Department of Medicine, Division of General Internal Medicine, University of California, San Francisco, California.

**Keywords:** diabetes mellitus, food insecurity, health care disparities, diabetes education, diet, food, nutrition

## Abstract

**Purpose:** Consuming a diet appropriate for management of diabetes mellitus (DM) is challenging, particularly for adults with food insecurity (FI). DM-related health care services are thought to support better dietary intake. In this study, we explored associations between DM-related health care utilization and dietary intake among FI adults with DM.

**Methods:** We used cross-sectional, baseline data (collected 2015–2016) from a trial designed to improve glycemic control among adult food pantry clients with DM. We examined intake of vegetables, fruit, sugar-sweetened beverages (SSBs), and desserts using the California Health Interview Survey dietary screener. We then examined adjusted associations between dietary intake and two components of DM-related health care utilization (<12 months vs. ≥12 months ago): self-reported visit to a health care provider for DM management and DM self-management education.

**Results:** Among 523 participants (mean hemoglobin A1c 9.8%; body mass index 34.6 kg/m^2^; 17.0% uninsured), vegetable intake was more frequent in those reporting recent utilization of health care providers for DM management and DSME-related services (*p*<0.01), compared with those with less recent use. There was no association between intake frequency of fruit or SSBs and utilization of either DM-related service. Participants more recently utilizing DSME-related services consumed desserts more frequently (*p*=0.02). Relationships persisted after controlling for DM duration, race/ethnicity, education, health insurance, location, medication adherence, and depression.

**Conclusions:** Among FI patients, DM-related services offered in clinical settings may more effectively increase vegetable consumption than decrease consumption of food and beverage items that can worsen glycemic control. Food pantry settings may provide an opportunity to reinforce dietary messaging.

## Introduction

Diabetes mellitus (DM) requires complex and multidimensional care, including diabetes education and self-management of diet, physical activity, and medications. Among these complex care needs, managing diet is the most challenging for many people, and is critically important for glycemic control, weight management, and comorbidity prevention.^1^ To support individuals with DM in consuming diets appropriate for glycemic management, both the American Diabetes Association (ADA) and the Centers for Disease Control and Prevention recommend that all patients actively engage with a health care team in self-management and treatment planning.^2,3^ As part of diabetes mellitus self-management education (DMSE), individuals with DM are encouraged to limit consumption of sugar-sweetened beverages (SSBs); limit foods with added sugar; and focus carbohydrate intake on healthier choices, including vegetables, fruit, and whole grains. Such dietary changes improve insulin sensitivity, glycemic control, and lipid profiles.^4,5^

While achieving these dietary changes can be difficult for all patients, food insecurity (FI) intensifies the challenges.^6^ FI refers to “limited or uncertain access to nutritionally adequate and safe food required for an active, healthy life.”^7^ About 37 million people in the United States live in food insecure households.^8^

For individuals with both FI and DM, coping strategies such as shifting dietary intake toward cheaper and more obesogenic foods and putting off medication purchases or clinic visits to pay for food may increase food access, but can worsen glycemic control.^9,10^ Food pantries and other emergency food services (e.g., soup kitchens) can also increase food access,^11,12^ but food available in these settings may be limited in variety, calorically dense, and/or poor in nutritional quality.^13,14^ Given the added burden FI places on DM self-management, individuals with both DM and FI may benefit from more intense support from health care providers and allied health professionals.^15,16^

Few studies have examined the relationship between utilization of DM-related health care services and dietary intake in the general population, and this relationship is particularly poorly understood among FI populations. Therefore, we sought to determine the extent to which utilization of health care provider services for DM and/or DSME is associated with improved dietary intake among adults with DM-seeking assistance at food pantries. We hypothesized that food pantry clients with DM more recently utilizing DM-related health care would report healthier dietary intake, including increased frequency of vegetables and fruit and reduced frequency of added sugar intake, compared with those with less recent or no utilization of DM-related health care services.

## Methods

### Setting and sample

This is a cross-sectional study using baseline data (collected from October 2015 to September 2016) from the Feeding America Intervention Trial for Health-Diabetes Mellitus (FAITH-DM) study. The design, methods, population, and results of FAITH-DM (NCT02569060) have previously been published.^17^ The research protocol for FAITH-DM was approved by the Western institutional review board with additional review (for data analysis only) by the University of California San Francisco and Urban Institute institutional review boards.^17^ All participants provided written informed consent before participation.

In brief, FAITH-DM was a 6-month randomized, waitlist-controlled trial performed at 27 food pantries affiliated with three food banks (Alameda County Community Food Bank, Oakland, CA; Gleaners Community Food Bank of Southeastern Michigan, Detroit, MI; and Houston Food Bank, Houston, TX). A waitlist control trial involves a group of participants first being assigned to a waiting list and then receiving intervention after the active treatment group, and can be performed when it may be unethical to deny participants access to treatment.^18^ A total of 568 individuals with DM participated in an intervention consisting of self-management support provided at the food pantry and provision of diabetes-appropriate food boxes every 2 weeks. The primary outcome was hemoglobin A1c (HbA1c).

Inclusion criteria were age ≥18 years; an onsite point-of-care (POC) HbA1c ≥7.5%; existing or new food pantry client; fluency in English and/or Spanish; a reliable mode of contact; and intention to remain in the study area for ≥12 months. Exclusion criteria included cognitive impairment, pregnancy or <6 weeks postpartum, and/or history of type 1 DM. For this analysis of baseline data, we considered all participants to have FI under the assumption that food pantry clients are currently, recently, or at high risk of FI. Indeed, studies have shown that most food pantry users report having FI.^11,12^ We excluded participants not diagnosed with DM until the day of trial enrollment, as these individuals would not have previously been subject to guidelines recommending DM-related health care utilization. In addition, we excluded participants not responding to survey items related to DM-related health care utilization.

### Measures

All measures for this secondary analysis were selected *a priori* based upon our hypothesis that food pantry clients with DM more recently utilizing DM-related health care would report healthier dietary intake frequency. We defined DM-related health care utilization as (1) visits with a medical provider (doctor or other health care professional) for DM management and/or (2) use of DSME-related services. All participants were first asked, “Is there one doctor or other health professional that you usually see for your diabetes? Do not include diabetes educators, dieticians, or foot and eye doctors.” To assess the last time a participant saw this provider, those who responded positively to this question were then asked, “When was the last time you saw this doctor or other health professional for your diabetes” *(<3 months; 3–6 months; 6–12 months; >12 months; never; don't know; refused*).

To assess the last time a participant used DSME-related services, all participants, including those responding negatively to having a medical provider for DM management, were asked, “When was the last time you saw a diabetes nurse educator or dietician or nutritionist for your diabetes? Do not include doctors or other health professionals” *(<3 months; 3–6 month; 6–12 months; >12 months; never; don't know; refused*).

We dichotomized DM-related health care utilization (visit with a medical provider for DM management and use of DSME-related services) into more recent utilization (within the last 12 months) and less recent utilization (>12 months ago or never). ADA recommendations for how often an adult with DM should visit a health care provider are individualized according to glycemic control and other factors.^19^ For DMSE, the recommended frequency is “at least annually.”^1^ We therefore dichotomized both utilization variables at 12 months for consistency, and performed sensitivity analyses examining the impact of dichotomizing the utilization variables at other cut-points: (1) within the last 6 months versus >6 months ago or never, and (2) any versus no reported utilization.

Our dietary intake variables included self-reported frequency of vegetables (green leafy lettuce or salad and/or other vegetables), fruit, SSBs (regular soda, fruit-flavored drinks, and 100% fruit juice), and desserts (baked and/or frozen). We did not include beans and potatoes with vegetable intake because these items contain higher carbohydrate loads and, in the case of potatoes, are high glycemic index; and the ADA emphasizes low-carbohydrate vegetables.^1,20^ Further, we considered 100% fruit juice as a SSB because the sugar content of these beverages may be similar to or higher than other SSBs.^21^

All dietary intake items were derived from the 2005 California Health Interview Survey (CHIS) dietary screener, a 10-item food frequency questionnaire that asks participants about food and beverage intake over the past month.^22^ We selected the above items from the CHIS dietary screener *a priori* because of our interest in specifically examining associations between health care utilization and intake of low-carbohydrate (nonstarchy) vegetables, fruit, and foods and beverages classically containing significant amounts of added sugar. All responses (times per day, week, or month) were converted into daily intake frequencies.

POC HbA1c testing was performed by trained food bank staff and volunteers. All other measures were obtained from the FAITH-DM baseline survey. Demographic characteristics included age, gender, self-reported height and weight (used to calculate body mass index), race/ethnicity (Caucasian/White, Black/African American, Hispanic/Latino, Native American, Asian or Pacific Islander, and Multi-racial), education level (some high school or less; high school graduate, General Education Development degree, some college, Associate of Arts, or technical school; college graduate and/or graduate degree), and presence of health insurance (including through current/former employer or union, purchased directly from insurance company, Medicare, Medicaid, Medical Assistance, Children's Health Insurance Program, TRICARE, Indian Health Services, and other).

We assessed medication nonadherence using four previously developed yes/no items: forgetting to take medications ever, not careful about taking medications at times, stopping taking medications when feeling better, and stopping taking medications if feel worse while taking them. Zero affirmatives to these questions represented the lowest level of nonadherence (highest adherence), and four affirmatives represented the highest level of nonadherence. Depressive symptoms were assessed using the Patient Health Questionnaire-8 (PHQ-8) scored from 0 to 24, with a score of 0–4 representing minimal depressive, 5–9 mild depressive, 10–14 moderate depressive, 15–19 moderately severe depressive, and 20–24 severe depressive symptoms per standard scoring procedures.^23^

### Data analysis

Data are reported as means with standard errors for continuous variables and as numbers with percentages for categorical variables. We compared sociodemographic characteristics of participants reporting more versus less recent DM-related health care utilization using *t*-tests for continuous and *χ*^2^ tests for categorical variables. We used *t*-tests to determine unadjusted associations between recentness of DM-related health care utilization and dietary intake frequencies, followed by multiple linear regression to examine these associations after adjusting for DM duration, race/ethnicity, education level, health insurance, medication adherence, PHQ-8 score, and food bank location. In these regression analyses, the model covariates were categorized as described above, and all covariates added to the regression models were selected given their potential role as confounders.^24–31^

Missing data for dietary intake items were <1% and for the model covariates, <2.5%. Statistical significance was based on a type I error rate of 0.05. Model fit was assessed by plotting residual versus fitted plots to assess for homoscedasticity. All statistical analyses were conducted using Stata 14.1 (StataCorp, College Station, TX).

## Results

### Sociodemographic characteristics

The data analysis included 523 of the 562 available participants (6.6% excluded because they were not diagnosed with DM until the day of survey administration; two participants excluded because they did not respond to DM-related health care utilization questions). Participants were predominantly female (68.0%), had a mean age of 54.8 years, and a mean PHQ-8 score consistent with mild depression (8.1±0.3). About 50% of participants reported consulting a medical provider for DM management and 30% reported utilizing DSME-related services within the last 12 months.

Sociodemographic characteristics comparing participants with more and less recent DM-related health care utilization are summarized in Table 1. Those reporting more recent DM-related health care utilization had a longer DM duration and a lower HbA1c compared with those reporting less recent use. Participants with insurance were less likely to have spent >12 months without seeing a medical provider for DM (*p*<0.01), but were not less likely to have received DSME-related services.

**Table 1.** Baseline Characteristics of Food Pantry Clients with Diabetes Mellitus Comparing More to Less Recent Diabetes Mellitus-Related Health Care Exposure

	Total (n=523)	DM medical provider exposure			DSME-related services exposure		
		>12 months ago/never (n=255)	Within the last 12 months (n=268)	p^a^	>12 months ago/never (n=364)	Within the last 12 months (n=159)	p^a^
Gender (% female)	355 (68.0%)	179 (70.2%)	176 (65.9%)	0.30	253 (69.7%)	102 (64.2%)	0.21
Age (mean±SE)	54.8±0.5	53.4±0.7	56.1±0.6	<0.01	55.1±0.6	54.1±0.8	0.34
Diabetes duration (years; mean±SE)	12.9±0.5	11.9±0.7	13.9±0.7	0.04	12.2±0.6	14.5±1.0	0.04
Body mass index (mean±SE)	34.6±0.4	34.4±0.6	34.7±0.5	0.73	34.7±0.5	34.3±0.7	0.58
A1c (%; mean±SE)	9.8±0.1	10.0±0.1	9.5±0.1	<0.01	9.9±0.1	9.5±0.1	0.04
Medication nonadherence score (mean±SE)	1.14±0.1	1.10±0.1	1.17±0.1	0.47	1.09±0.1	1.23±0.1	0.24
Depression (PHQ-8; mean±SE)	8.1±0.3	8.7±0.4	7.6±0.4	0.04	8.2±0.3	8.0±0.5	0.77
Food bank location							
Detroit	175 (33.5%)	127 (49.8%)	48 (17.9%)	<0.01	145 (39.8%)	30 (18.9%)	<0.01
Houston	240 (45.9%)	79 (31.0%)	161 (60.1%)		156 (42.9%)	84 (52.8%)	
Oakland	108 (20.7%)	49 (19.2%)	59 (22.0%)		63 (17.3%)	45 (28.3%)	
Race/ethnicity							
Caucasian/White	69 (13.2%)	38 (14.9%)	31 (11.6%)	0.27	57 (15.7%)	12 (7.6%)	0.05
Black/African American	176 (33.7%)	92 (36.1%)	84 (31.3%)		123 (33.8%)	53 (33.3%)	
Latino/Hispanic	262 (50.1%)	119 (46.7%)	143 (53.4%)		172 (47.3%)	90 (56.6%)	
Other^b^	16 (3.1%)	6 (2.4%)	10 (3.7%)		12 (3.3%)	4 (2.5%)	
Education							
Some high school or less	241 (46.2%)	112 (43.9%)	129 (48.3%)	0.04	164 (45.1%)	77 (48.7%)	0.43
HS Grad/GED/some college/AA/Tech	242 (46.4%)	130 (51.0%)	112 (42.0%)		175 (48.1%)	67 (42.4%)	
College grad/grad degree	39 (7.5%)	13 (5.1%)	26 (9.7%)		25 (6.9%)	14 (8.9%)	
Uninsured (%)^c^	87 (17.0%)	54 (21.6%)	33 (12.6%)	<0.01	62 (17.4%)	25 (1 5.9%)	0.68

^a^ *t*-Test except race/ethnicity and education (*χ*^2^).

^b^ Includes Native American, Asian or Pacific Islander, multiracial, and other.

^c^ Including insurance through current/former employer or union, purchased directly from insurance company, Medicare, Medicaid, Medical Assistance, Children's Health Insurance Program, TRICARE, Indian Health Services, and other.

AA, Associate of Arts; DM, diabetes mellitus; DSME, diabetes self-management education; GED, general education development; HS, high school; PHQ-8, Patient Health Questionnaire-8 (depression screener); SE, standard error.

In sensitivity analyses (Supplementary Tables S1 and S2), similar results were observed when DM-related health care utilization was dichotomized at 6 months, and at ever (vs. never) except for the following: (1) DM duration, PHQ-8 scores, and education level were not statistically significantly different between groups when visits with a medical provider for DM management were dichotomized at 6 months; and (2) DM duration was not statistically significantly different between groups when DSME-related service use was dichotomized at 6 months.

### Dietary intake

Associations between DM-related health care utilization and dietary intake frequency are summarized in Table 2. In the unadjusted analyses, vegetable consumption frequency was greater in those reporting more recent utilization of medical providers for DM management (1.2 times/day vs. 0.9 times/day; *p*<0.01) and DSME-related services (1.2 times/day vs. 1.0 times/day; *p*<0.01) compared with those reporting less recent use. There was no difference between those with more or less recent utilization of either DM-related health care service in fruit or SSB consumption frequency. Participants more recently utilizing DSME-related services consumed desserts more frequently compared with those with less recent use (*p*=0.02). After adjusting for DM duration, race/ethnicity, education level, health insurance status, medication adherence, PHQ-8 score, and food bank location, all of the statistically significant associations between DM-related health care utilizations and dietary consumption frequencies observed in the unadjusted analyses persisted.

**Table 2.** Associations Between Diabetes Mellitus Health Care Visits and Dietary Consumption Frequency Among Food Pantry Clients with Diabetes Mellitus

	Dietary consumption frequency—unadjusted (times/day)			Dietary consumption frequency—adjusted (times/day)^a^		
	>12 months ago/never	Within the last 12 months	p	>12 months ago/never	Within the last 12 months	p
DM medical provider exposure						
Sugar-sweetened beverages (mean±SE)^b^	0.80±0.06	0.68±0.06	0.18	0.76±0.07	0.69±0.07	0.53
Desserts (mean±SE)^c^	0.33±0.03	0.34±0.03	0.70	0.31±0.04	0.38±0.03	0.25
Vegetables (mean±SE)^d^	0.89±0.06	1.21±0.06	<0.01	0.90±0.07	1.22±0.07	<0.01
Fruit (mean±SE)	0.74±0.78	0.83±0.73	0.16	0.73±0.05	0.86±0.05	0.08
DSME-related services exposure						
Sugar-sweetened beverages (mean±SE)^a^	0.70±0.04	0.82±0.10	0.20	0.67±0.06	0.84±0.09	0.10
Desserts (mean±SE)^b^	0.30±0.02	0.41±0.05	0.02	0.31±0.03	0.43±0.04	0.03
Vegetables (mean±SE)^c^	0.97±0.05	1.24±0.09	<0.01	1.00±0.06	1.24±0.09	0.02
Fruit (mean±SE)	0.76±0.04	0.84±0.06	0.27	0.79±0.04	0.81±0.06	0.81

^a^ Adjusted for duration of diabetes, race/ethnicity, education, health insurance status, study site, and medication adherence, and depression (PHQ-8).

^b^ Includes nondiet soda, nondiet flavored drinks, and 100% fruit juice.

^c^ Includes baked and frozen desserts.

^d^ Includes greens and other vegetables.

In sensitivity analyses (Supplementary Table S3), similar results were obtained when DM-related health care utilizations were dichotomized at 6 months and by ever (vs. never) utilizing these services, except for the following: (1) fruit consumption frequency was greater in those reporting ever (vs. never) receiving DSME-related services (0.9 times/day vs. 0.7 times/day; *p*=0.02) in unadjusted analysis, (2) vegetable consumption frequency was not statistically significantly between those receiving DSME-related services within the last 6 months versus >6 months ago in adjusted analysis, and (3) dessert consumption was not statistically significantly different between those receiving DSME-related services more or less recently for both sensitivity analyses.

## Discussion

In this study of U.S. food pantry clients with DM, more recent utilization of a medical provider for DM management and/or DSME-related services were both associated with increased vegetable consumption frequency. However, intake frequency of SSB and desserts was not associated with utilization of either DM-related health care service. These relationships persisted after controlling for sociodemographic characteristics, and largely persisted in sensitivity analyses, suggesting the robustness of these findings.

There are several possibilities to consider for why recent DM-related health care utilization was associated with increased vegetable consumption frequency. It may be that use of DM-related services motivates an individual to increase dietary consumption of healthy foods such as vegetables. This hypothesis is consistent with the theory of change underlying most DM-related services. Indeed, studies have shown that DSME is associated with improved DM knowledge, self-care behaviors, and increased likelihood of following best practice treatment recommendations.^32–36^ Given the cross-sectional nature of this study however, it is also possible that individuals consuming vegetables more often seek DM care, or have fewer barriers to accessing DM-related health care services. The finding that participants who reported seeing a medical provider for DM management were also more likely to be insured supports this theory.

Contrary to our hypothesis, those more recently utilizing DM-related health care services did not report decreased SSB or dessert intake frequency. There are several potential explanations for this finding. First, the impact that DM-related health care services have on added sugar consumption has not been well studied and is therefore unclear, despite being one of the primary intended goals of DSME. While DSME is associated with improved DM-related outcomes,^1^ it is possible that such interventions have more impact on improving other DM-related lifestyle modifications (e.g., increasing medication compliance, promoting physical activity) and less effect on added sugar intake itself.

Second, the food environment may overwhelm nutritional messaging originating in the clinical setting. Food pantries are predominantly situated in low-income neighborhoods and cater to low-income clients.^12^ Low-income neighborhoods have a higher prevalence of fast food restaurants and convenience stores (so called “food swamps”) compared with high-income areas.^37^ In addition, although the charitable feeding system has dramatically increased the nutrient content of available food recently,^38,39^ it was originally designed for the distribution of shelf-stable food donations that are generally calorically dense and poorer in nutritional quality. Chronic exposure to poorer dietary options may contribute to dietary choices. Indeed, environmental cues for sugar consumption in low-income neighborhoods and/or in food pantries, where these cues may be more pervasive^40^ and healthier dietary options less available,^39^ may supersede messaging from the health care setting to avoid these unhealthy options.

Third, it is possible that messaging from traditional health care settings to avoid added sugar is overall less successful than messaging to increase intake of healthier dietary options. In particular, in the case of FI it may be that the appeal of calorically dense foods makes countermessaging more difficult, as substitutions for these foods with healthier alternatives are often cost prohibitive.^41^

Strengths of this study include a large multiethnic cohort with participants from three food banks representing different demographic locations across the United States. However, our results must be interpreted within the context of a number of limitations. As this is a cross-sectional study, we cannot be certain that utilization of DM-related health care services is causally related to dietary intake. The CHIS dietary screener does not allow for direct determination of portion sizes, and self-reported measures of health care utilization were not objectively verified. Further, the CHIS dietary screener does not specifically ask about intake of presweetened teas, sports drinks, and energy drinks, which may also impact glycemic control similar to other SSBs. Thus, our findings should be considered preliminary, and future studies including additional measures of intake, such as food diaries and 24-h dietary recalls, are needed for validation.^42^

Moreover, our results may not be generalizable outside of the food pantry setting or to individuals with type 1 DM. Finally, nutritional knowledge was not specifically assessed. While the overall association between nutritional knowledge and dietary intake is only weakly positive,^43^ it is possible that dietary knowledge plays a more influential role in the decision to consume a healthier diet than the encounter with a DM health care provider itself. Further studies should explore the role that nutritional knowledge has in mediating the relationship between DM-related health care utilization and dietary intake, including among those with FI.

## Conclusions

Among food pantry users, more recent utilization of DM-related health care was associated with increased vegetable consumption frequency, but not with decreased SSB or dessert consumption frequency. Among highly vulnerable patients, DM-related services traditionally offered in the clinical setting may be more effective at increasing consumption of healthier items such as vegetables than decreasing the consumption of less healthy items that can worsen glycemic control. The food pantry setting may, therefore, provide an additional opportunity to reinforce healthy dietary intake messaging.

## Supplementary Material

Supplemental data

Supplemental data

Supplemental data

## Abbreviations Used

ADA: American Diabetes Association
BMI: body mass index
CHIS: California Health Interview Survey
DM: diabetes mellitus
DMSE: DM self-management education
FAITH-DM: Feeding America Intervention Trial for Health-Diabetes Mellitus
FI: food insecurity
HbA1c: hemoglobin A1c
PHQ-8: Patient Health Questionnaire-8
POC: point of care
SE: standard error
SSBs: sugar-sweetened beverages
