# Supplementary material for: Diabetes-Related Health Care Utilization and Dietary Intake Among Food Pantry Clients
Source: Health Equity. 2019 Dec 17;3(1):644–51. doi: 10.1089/heq.2019.0102 (PMC6921093; doi:10.1089/heq.2019.0102)
Supplement: Supplemental data [file Supp_Table1.pdf]

Supplementary Data

Supplementary Table S1. Baseline Characteristics of Food Pantry Clients with Diabetes Mellitus Comparing More to Less Recent Diabetes Mellitus Medical Provider Exposure—Sensitivity Analyses

		Within last 12 months			Within last 6 months			Never versus ever		
DM medical provider exposure	Total ($n=523$)	> 12 months ago/ never ($n=255$)	Within the last 12 months ($n=268$)	p[a]	> 6 months ago/ never ($n=266$)	Within the last 6 months ($n=257$)	p[a]	Never ($n=241$)	Ever ($n=282$)	p[a]
Gender (% female)	355 (68.0%)	179 (70.2%)	176 (65.9%)	0.30	183 (68.8%)	172 (67.2%)	0.69	167 (69.3%)	188 (66.9%)	0.56
Age (mean ± SE)	54.8 ± 0.5	53.4 ± 0.7	56.1 ± 0.6	< 0.01	53.4 ± 0.7	56.2 ± 0.7	< 0.01	53.7 ± 0.7	55.7 ± 0.6	0.05
Diabetes duration (years; mean ± SE)	12.9 ± 0.5	11.9 ± 0.7	13.9 ± 0.7	0.04	12.1 ± 0.7	13.8 ± 0.7	0.11[b]	11.8 ± 0.7	13.9 ± 0.7	0.04
BMI (mean ± SE)	34.6 ± 0.4	34.4 ± 0.6	34.7 ± 0.5	0.73	34.5 ± 0.6	34.7 ± 0.5	0.75	34.5 ± 0.6	34.7 ± 0.5	0.80
A1c (%; mean ± SE)	9.8 ± 0.1	10.0 ± 0.1	9.5 ± 0.1	< 0.01	10.0 ± 0.1	9.5 ± 0.1	< 0.01	10.0 ± 0.1	9.5 ± 0.1	< 0.01
Medication nonadherence score (mean ± SE)	1.14 ± 0.1	1.10 ± 0.1	1.17 ± 0.1	0.47	1.11 ± 0.1	1.17 ± 0.1	0.56	1.10 ± 0.1	1.19 ± 0.1	0.28
Depression (PHQ-8; mean ± SE)	8.1 ± 0.3	8.7 ± 0.4	7.6 ± 0.4	0.04	8.5 ± 0.4	7.8 ± 0.4	0.23[b]	8.8 ± 0.4	7.6 ± 0.4	0.03
Food bank location										
Detroit	175 (33.5%)	127 (49.8%)	48 (17.9%)	< 0.01	127 (47.7%)	48 (18.7%)	< 0.01	124 (51.5%)	51 (18.1%)	< 0.01
Houston	240 (45.9%)	79 (31.0%)	161 (60.1%)		89 (33.5%)	151 (58.8%)		71 (29.5%)	169 (59.9%)	
Oakland	108 (20.7%)	49 (19.2%)	59 (22.0%)		50 (18.8%)	58 (22.6%)		46 (19.1%)	62 (22.0%)	
Race/ethnicity										
Caucasian/White	69 (13.2%)	38 (14.9%)	31 (11.6%)	0.27	40 (15.0%)	29 (11.3%)	0.18	36 (14.9%)	33 (11.7%)	0.15
Black/African American	176 (33.7%)	92 (36.1%)	84 (31.3%)		97 (36.5%)	79 (30.7%)		87 (36.1%)	89 (31.6%)	
Latino/Hispanic	262 (50.1%)	119 (46.7%)	143 (53.4%)		122 (45.9%)	140 (54.5%)		114 (47.3%)	148 (52.5%)	
Other[c]	16 (3.1%)	6 (2.4%)	10 (3.7%)		7 (2.6%)	9 (3.5%)		4 (1.7%)	12 (4.3%)	
Education										
Some high school or less	241 (46.2%)	112 (43.9%)	129 (48.3%)	0.04	116 (43.6%)	125 (48.8%)	0.12[b]	108 (44.8%)	133 (47.3%)	0.03
HS grad/GED/some college/AA/Tech	242 (46.4%)	130 (51.0%)	112 (42.0%)		134 (50.4%)	108 (42.2%)		122 (50.6%)	120 (42.7%)	
College grad/grad degree	39 (7.5%)	13 (5.1%)	26 (9.7%)		16 (6.0%)	23 (9.0%)		11 (4.6%)	28 (10.0%)	
Uninsured (%)[d]	87 (17.0%)	54 (21.6%)	33 (12.6%)	< 0.01	57 (21.8%)	30 (11.9%)	< 0.01	50 (21.2%)	37 (13.4%)	0.02

Comparing impact of dichotomizing visits with a medical provider for DM management at various cut-points (within the last 6 months versus >6 months ago or never; never versus ever).

[a] *t*-Test except race/ethnicity and education (χ^2).

[b] Statistical significance varies from baseline comparator (within last 12 months vs. >12 months ago or never).

[c] Includes Native American, Asian or Pacific Islander, multiracial, and other.

[d] Including insurance through current/former employer or union, purchased directly from insurance company, Medicare, Medicaid, Medical Assistance, CHIP, TRICARE, Indian Health Services, and other.

AA, Associate of Arts; DM, diabetes mellitus; GED, general education development; HS, high school; PHQ-8, Patient Health Questionnaire-8 (depression screener); SE, standard error.
