# Supplementary material for: Diabetes-Related Health Care Utilization and Dietary Intake Among Food Pantry Clients
Source: Health Equity. 2019 Dec 17;3(1):644–51. doi: 10.1089/heq.2019.0102 (PMC6921093; doi:10.1089/heq.2019.0102)
Supplement: Supplemental data [file Supp_Table2.pdf]

Supplementary Table S2. Baseline Characteristics of Food Pantry Clients with Diabetes Mellitus Comparing More to Less Recent Diabetes Self-Management Education-Related Health Care Exposure—Sensitivity Analyses

		Within last 12 months			Within last 6 months			Never versus ever		
DSME-related services exposure	Total ($n=523$)	>12 months ago/never ($n=364$)	Within the last 12 months ($n=159$)	p[a]	>6 months ago/never ($n=394$)	Within the last 6 months ($n=129$)	p[a]	Never ($n=227$)	Ever ($n=296$)	p[a]
Gender (% female)	355 (68.0%)	253 (69.7%)	102 (64.2%)	0.21	273 (69.5%)	82 (63.6%)	0.21	158 (69.9%)	197 (66.6%)	0.42
Age (mean ± SE)	54.8 ± 0.5	55.1 ± 0.6	54.1 ± 0.8	0.34	54.8 ± 0.6	54.6 ± 0.9	0.84	55.1 ± 0.8	54.5 ± 0.6	0.53
Diabetes duration (years; mean ± SE)	12.9 ± 0.5	12.2 ± 0.6	14.5 ± 1.0	0.04	12.5 ± 0.6	14.2 ± 1.1	0.14[b]	11.7 ± 0.8	13.9 ± 0.7	0.04
BMI (mean ± SE)	34.6 ± 0.4	34.7 ± 0.5	34.3 ± 0.7	0.58	34.8 ± 0.5	33.8 ± 0.8	0.26	34.1 ± 0.5	34.9 ± 0.6	0.31
A1c (%; mean ± SE)	9.8 ± 0.1	9.9 ± 0.1	9.5 ± 0.1	0.04	9.8 ± 0.1	9.5 ± 0.2	0.04	10.0 ± 0.1	9.6 ± 0.1	< 0.01
Medication nonadherence score (mean ± SE)	1.14 ± 0.1	1.09 ± 0.1	1.23 ± 0.1	0.24	1.09 ± 0.1	1.28 ± 0.1	0.11	1.08 ± 0.1	1.18 ± 0.1	0.32
Depression (PHQ-8; mean ± SE)	8.1 ± 0.3	8.2 ± 0.3	8.0 ± 0.5	0.77	8.2 ± 0.3	7.8 ± 0.5	0.52	7.9 ± 0.4	8.3 ± 0.3	0.50
Food bank location										
Detroit	175 (33.5%)	145 (39.8%)	30 (18.9%)	< 0.01	150 (38.1%)	25 (19.4%)	< 0.01	105 (46.3%)	70 (23.7%)	< 0.01
Houston	240 (45.9%)	156 (42.9%)	84 (52.8%)		171 (43.4%)	69 (53.5%)		97 (42.7%)	143 (48.3%)	
Oakland	108 (20.7%)	63 (17.3%)	45 (28.3%)		73 (18.5%)	35 (27.1%)		25 (11.0%)	83 (28.0%)	
Race/ethnicity										
Caucasian/White	69 (13.2%)	57 (15.7%)	12 (7.6%)	0.05	61 (15.5%)	8 (6.2%)	0.05	39 (17.2%)	30 (10.1%)	0.05
Black/African American	176 (33.7%)	123 (33.8%)	53 (33.3%)		130 (33.0%)	46 (35.7%)		78 (34.4%)	98 (33.1%)	
Latino/Hispanic	262 (50.1%)	172 (47.3%)	90 (56.6%)		190 (48.2%)	72 (55.8%)		106 (46.7%)	156 (52.7%)	
Other[c]	16 (3.1%)	12 (3.3%)	4 (2.5%)		13 (3.3%)	3 (2.3%)		4 (1.8%)	12 (4.1%)	
Education										
Some high school or less	241 (46.2%)	164 (45.1%)	77 (48.7%)	0.43	180 (45.7%)	61 (44.7%)	0.89	113 (49.8%)	128 (43.4%)	0.09
HS grad/GED/some college/AA/Tech	242 (46.4%)	175 (48.1%)	67 (42.4%)		185 (47.0%)	57 (44.5%)		103 (45.4%)	139 (47.1%)	
College grad/grad degree	39 (7.5%)	25 (6.9%)	14 (8.9%)		29 (7.4%)	10 (7.8%)		11 (4.9%)	28 (9.5%)	
Uninsured (%)[d]	87 (17.0%)	62 (17.4%)	25 (15.9%)	0.68	70 (18.1%)	17 (13.4%)	0.22	31 (14.0%)	56 (19.2%)	0.11

Comparing impact of dichotomizing DSME-related service exposure at various cut-points (within the last 6 months versus >6 months ago or never; never vs. ever).

[a] *t*-Test except race/ethnicity and education (χ^2).

[b] Statistical significance varies from baseline comparator (within last 12 months vs. >12 months ago or never).

[c] Includes Native American, Asian or Pacific Islander, multiracial, and other.

[d] Including insurance through current/former employer or union, purchased directly from insurance company, Medicare, Medicaid, Medical Assistance, CHIP, TRICARE, Indian Health Services, and other.
