# Supplementary material for: Diabetes-Related Health Care Utilization and Dietary Intake Among Food Pantry Clients
Source: Health Equity. 2019 Dec 17;3(1):644–51. doi: 10.1089/heq.2019.0102 (PMC6921093; doi:10.1089/heq.2019.0102)
Supplement: Supplemental data [file Supp_Table3.pdf]

Supplementary Table S3. Associations Between Diabetes Mellitus Health Care Visits and Dietary Consumption Frequency Among Food Pantry Clients with Diabetes Mellitus—Sensitivity Analyses

	Dietary consumption frequency—unadjusted (times/day)			Dietary consumption frequency—adjusted (times/day)[a]		
	>12 months ago/never	Within the last 12 months	*p*	>12 months ago/never	Within the last 12 months	*p*
DM medical provider exposure						
Sugar-sweetened beverages (mean ± SE)[b]	0.80 ± 0.06	0.68 ± 0.06	0.18	0.76 ± 0.07	0.69 ± 0.07	0.49
Desserts (mean ± SE)[c]	0.33 ± 0.03	0.34 ± 0.03	0.70	0.31 ± 0.04	0.38 ± 0.03	0.25
Vegetables (mean ± SE)[d]	0.89 ± 0.06	1.21 ± 0.06	< 0.01	0.90 ± 0.07	1.21 ± 0.07	< 0.01
Fruit (mean ± SE)	0.74 ± 0.78	0.83 ± 0.73	0.16	0.73 ± 0.05	0.86 ± 0.05	0.07
DSME-related services exposure						
Sugar-sweetened beverages (mean ± SE)[b]	0.70 ± 0.04	0.82 ± 0.10	0.20	0.67 ± 0.06	0.84 ± 0.08	0.10
Desserts (mean ± SE)[c]	0.30 ± 0.02	0.41 ± 0.05	0.02	0.31 ± 0.03	0.43 ± 0.04	0.03
Vegetables (mean ± SE)[d]	0.97 ± 0.05	1.24 ± 0.09	< 0.01	1.00 ± 0.06	1.23 ± 0.08	0.02
Fruit (mean ± SE)	0.76 ± 0.04	0.84 ± 0.06	0.27	0.79 ± 0.04	0.81 ± 0.06	0.83
	>6 months ago/never	Within the last 6 months	*p*	>6 months ago/never	Within the last 6 months	*p*
DM medical provider exposure						
Sugar-sweetened beverages (mean ± SE)[b]	0.81 ± 0.06	0.67 ± 0.06	0.11	0.76 ± 0.07	0.69 ± 0.07	0.49
Desserts (mean ± SE)[c]	0.32 ± 0.03	0.35 ± 0.03	0.56	0.31 ± 0.04	0.39 ± 0.04	0.16
Vegetables (mean ± SE)[d]	0.89 ± 0.05	1.22 ± 0.06	< 0.01	0.91 ± 0.07	1.22 ± 0.07	< 0.01
Fruit (mean ± SE)	0.74 ± 0.05	0.83 ± 0.05	0.14	0.73 ± 0.05	0.85 ± 0.05	0.11
DSME-related services exposure						
Sugar-sweetened beverages (mean ± SE)[b]	0.72 ± 0.05	0.78 ± 0.10	0.54	0.69 ± 0.06	0.81 ± 0.09	0.28
Desserts (mean ± SE)[c]	0.32 ± 0.02	0.39 ± 0.05	0.19[e]	0.33 ± 0.03	0.40 ± 0.05	0.18[e]
Vegetables (mean ± SE)[d]	1.00 ± 0.05	1.21 ± 0.09	0.03	1.02 ± 0.05	1.21 ± 0.09	0.09[e]
Fruit (mean ± SE)	0.77 ± 0.04	0.83 ± 0.06	0.39	0.80 ± 0.04	0.79 ± 0.07	0.94
	Never	Ever	*p*	Never	Ever	*p*
DM medical provider exposure						
Sugar-sweetened beverages (mean ± SE)[b]	0.80 ± 0.06	0.68 ± 0.06	0.15	0.78 ± 0.07	0.68 ± 0.06	0.35
Desserts (mean ± SE)[c]	0.33 ± 0.03	0.34 ± 0.03	0.84	0.32 ± 0.04	0.37 ± 0.03	0.36
Vegetables (mean ± SE)[d]	0.89 ± 0.06	1.20 ± 0.06	< 0.01	0.89 ± 0.07	1.21 ± 0.06	< 0.01
Fruit (mean ± SE)	0.73 ± 0.05	0.83 ± 0.04	0.17	0.72 ± 0.05	0.86 ± 0.05	0.06
DSME-related services exposure						
Sugar-sweetened beverages (mean ± SE)[b]	0.72 ± 0.05	0.75 ± 0.07	0.82	0.66 ± 0.07	0.77 ± 0.06	0.32
Desserts (mean ± SE)[c]	0.29 ± 0.03	0.37 ± 0.03	0.07[e]	0.30 ± 0.04	0.38 ± 0.03	0.08[e]
Vegetables (mean ± SE)[d]	0.91 ± 0.06	1.16 ± 0.06	< 0.01	0.92 ± 0.07	1.18 ± 0.06	< 0.01
Fruit (mean ± SE)	0.69 ± 0.05	0.85 ± 0.05	0.02[e]	0.75 ± 0.05	0.83 ± 0.05	0.32

Comparing impact of dichotomizing DM-related health care exposure at various cut-points (within the last 6 months versus >6 months ago or never; never versus ever).

[a]Adjusted for duration of diabetes, race/ethnicity, education, health insurance status, study site, and medication adherence, and depression (PHQ-8).

[b]Includes nondiet soda, nondiet flavored drinks, and 100% fruit juice.

[c]Includes baked and frozen desserts.

[d]Includes greens and other vegetables.

[e]Statistical significance varies from baseline comparator (within last 12 months versus >12 months ago or never).
